# C1QB-Mediated Immunopathology in a Murine Malaria Model: A Multi-Omics Validation for Diagnostic and Therapeutic Targeting

**DOI:** 10.3390/ijms27167459

**Published:** 2026-08-20

**Authors:** Yue Xie, Jieying Zheng, Jianan Zhao, Kaixuan Zhai, Fanchao Zhou, Wen Ye, Rong Xiang, Changsheng Deng, Jiafu Jiang

**Affiliations:** 1 Artemisinin Research Center, Guangzhou University of Chinese Medicine, Guangzhou 510080, China; 2 State Key Laboratory of Pathogen and Biosecurity, Beijing Institute of Microbiology and Epidemiology, Academy of Military Medical Sciences, Beijing 100071, China; 3 Department of Cardiovascular Sciences, Temple University Philadelphia, Philadelphia, PA 19122-6096, USA; aaronliver@163.com

**Keywords:** *Plasmodium falciparum*, diagnostic biomarker, transcriptomics, C1q

## Abstract

Malaria pathogenesis involves complex immunopathological mechanisms that hinder early diagnosis and effective treatment. This study integrates multi-omics data and experimental models to identify host-derived biomarkers and elucidate their functional roles. By combining human transcriptomic datasets, weighted gene co-expression network analysis (WGCNA), and machine learning (LASSO, SVM, RF), we identified C1QB as a key hub gene. In human data, C1QB was significantly upregulated in both training and validation cohorts (AUC 0.983 and 0.970). Single-gene GSEA and immune infiltration analyses linked C1QB to apoptosis, inflammation, and altered immune cell composition, including increased activated dendritic cells and neutrophils, and decreased naïve B cells and CD8^+^ T cells. In a murine malaria model (*Plasmodium berghei* ANKA), C1QB expression rose as early as day one post-infection, preceding detectable parasitemia. Immunohistochemistry revealed C1QB accumulation in the liver and spleen. Single-cell RNA sequencing in the murine model confirmed monocyte-predominant expression, and scTenifoldKnk analysis suggested its role in immune regulation. Crucially, inhibiting C1q in mice via antibody intervention alleviated malaria-induced inflammation, tissue damage, and apoptosis, indicating that C1QB/C1q actively contributes to immunopathology. AI-based drug prediction and molecular docking further supported its therapeutic potential. Collectively, our findings establish C1QB as a dual biomarker and pathogenic driver in malaria, with diagnostic and therapeutic implications. Further studies are required to validate direct target engagement and clarify upstream regulatory mechanisms.

## 1. Introduction

Malaria, a life-threatening disease caused by *Plasmodium* parasites, remains a major global health and economic burden, especially in tropical and subtropical regions [1]. The infection progresses through distinct phases—an asymptomatic liver stage followed by a symptomatic blood stage—each linked to specific pathological changes [2]. *Plasmodium* triggers a potent immune-inflammatory response, which is essential for parasite control but can also cause excessive inflammation and tissue damage if dysregulated [3,4]. Understanding these host-directed mechanisms is therefore key to developing better diagnostic and therapeutic approaches against malaria.

The complement system is a critical component of innate immunity, defending against infections through pathogen opsonization, inflammatory cell recruitment, and direct microbial lysis [5]. However, its activity is a double-edged sword: while essential for clearing pathogens, excessive or dysregulated activation can drive immunopathology and tissue damage [6,7]. Within this cascade, C1q—whose expression is notably regulated by the C1QB gene—acts as the initiator of the classical pathway. Despite its established role in immunity, systematic studies on C1q/C1QB in malaria remain limited [8,9]. Elucidating how C1q/C1QB-driven pathways influence malaria immunopathology may therefore reveal new diagnostic and therapeutic opportunities.

Current clinical diagnosis of malaria relies primarily on blood smear microscopy, rapid diagnostic tests (RDTs), and nucleic acid amplification. However, existing tools face clear limitations in achieving early, sensitive, and widely accessible diagnosis [10]. Recently, host-derived biomarkers have attracted growing interest in infectious disease diagnostics [11]. Unlike direct pathogen detection, these markers reflect the dynamic host immune response to infection. Immune molecules have been linked to disease presence and severity [12], underscoring their potential as complementary diagnostic tools, especially for tracking disease progression. Although previous studies have reported numerous host transcriptome changes and immune response genes associated with malaria, these findings are often influenced by regional differences, infection stages, sample heterogeneity, and research strategies, limiting their stability and clinical translational value [13,14]. Currently, there is still a lack of host-derived diagnostic markers that can be stably detected across different cohorts, dynamically reflect infection progression, and closely relate to malaria immune-pathological processes. Moreover, most existing studies focus on traditional bulk transcriptome analyses, with insufficient integration of machine learning screening, immune cell infiltration, single-cell localization, dynamic expression and functional validation. Therefore, it is difficult to systematically reveal the biological roles and translational potential of key genes in the development of malaria. Thus, identifying stable key genes that possess both diagnostic value and immunoregulatory significance remains an important need in current malaria research. Based on this, the present study integrates large-scale transcriptome data from multiple regions and combines WGCNA and machine learning, immune infiltration analysis, single-cell sequencing, dynamic validation in animals, and functional intervention experiments to systematically screen and validate key hub genes associated with malaria.

## 2. Results

### 2.1. C1QB Is Upregulated in Malaria and Is a Diagnostic Biomarker

**Figure 1:**
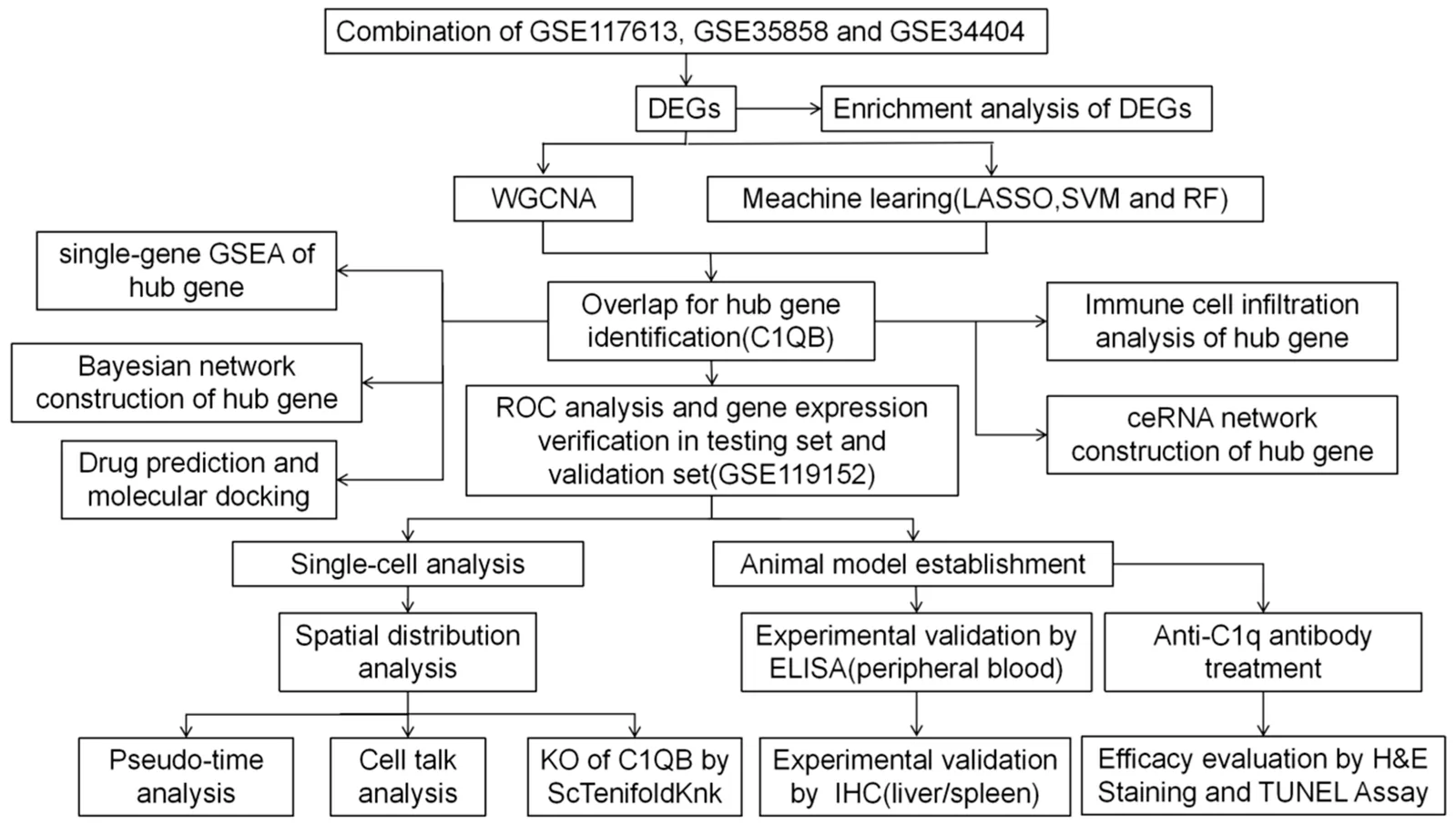
Flowchart of the study.

**Figure 2:**
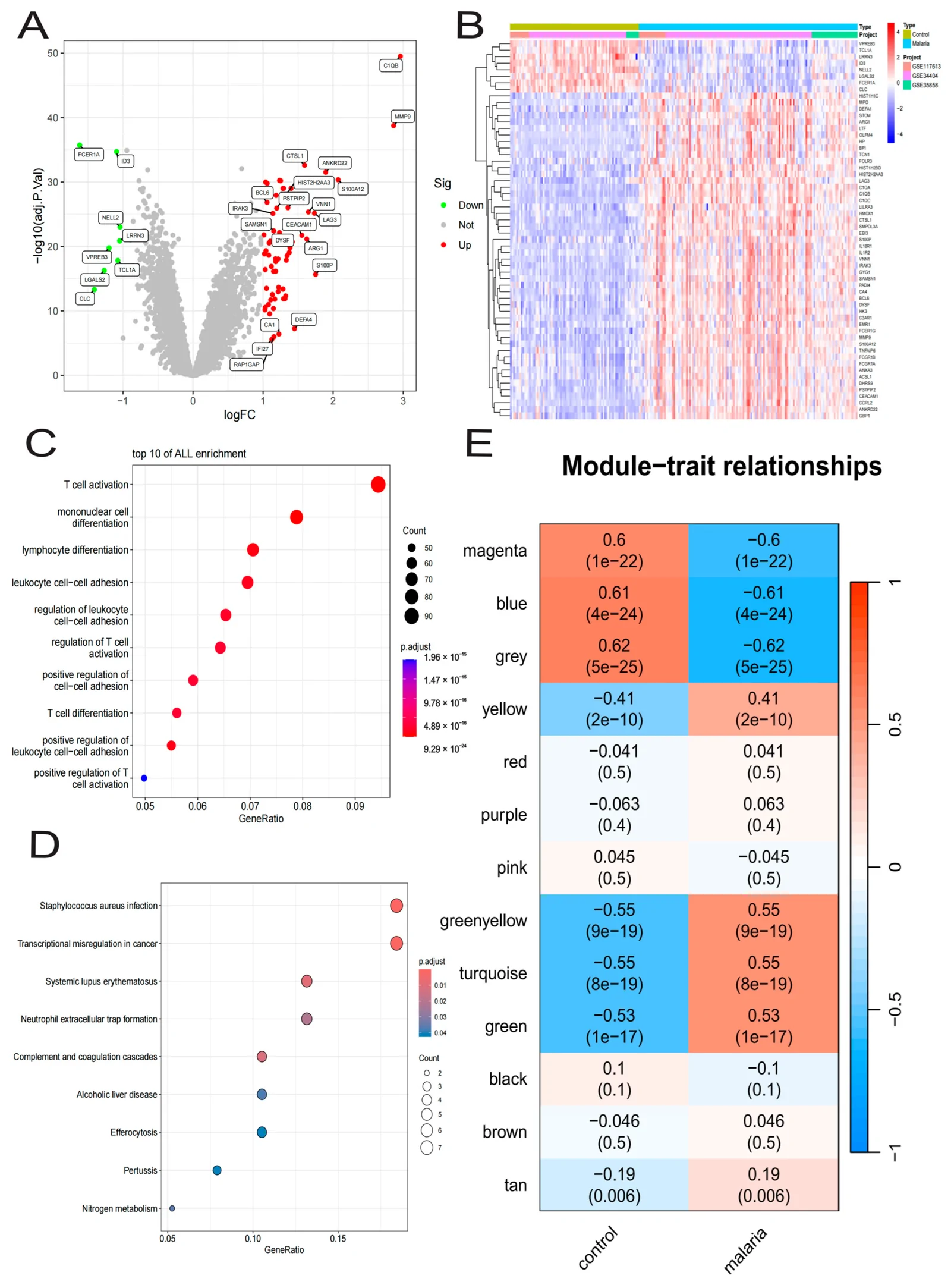
DEG analysis to identify malaria from merged training set. (**A**) DEG volcano plot. (**B**) Cluster heatmap of DEGs in the malaria group and the control group. (**C**) GO enrichment analysis of DEGs. (**D**) KEGG enrichment analysis of DEGs. (**E**) Module–trait relationships of WGCNA. Each cell contains the corresponding correlation and *p*-value.

**Figure 3:**
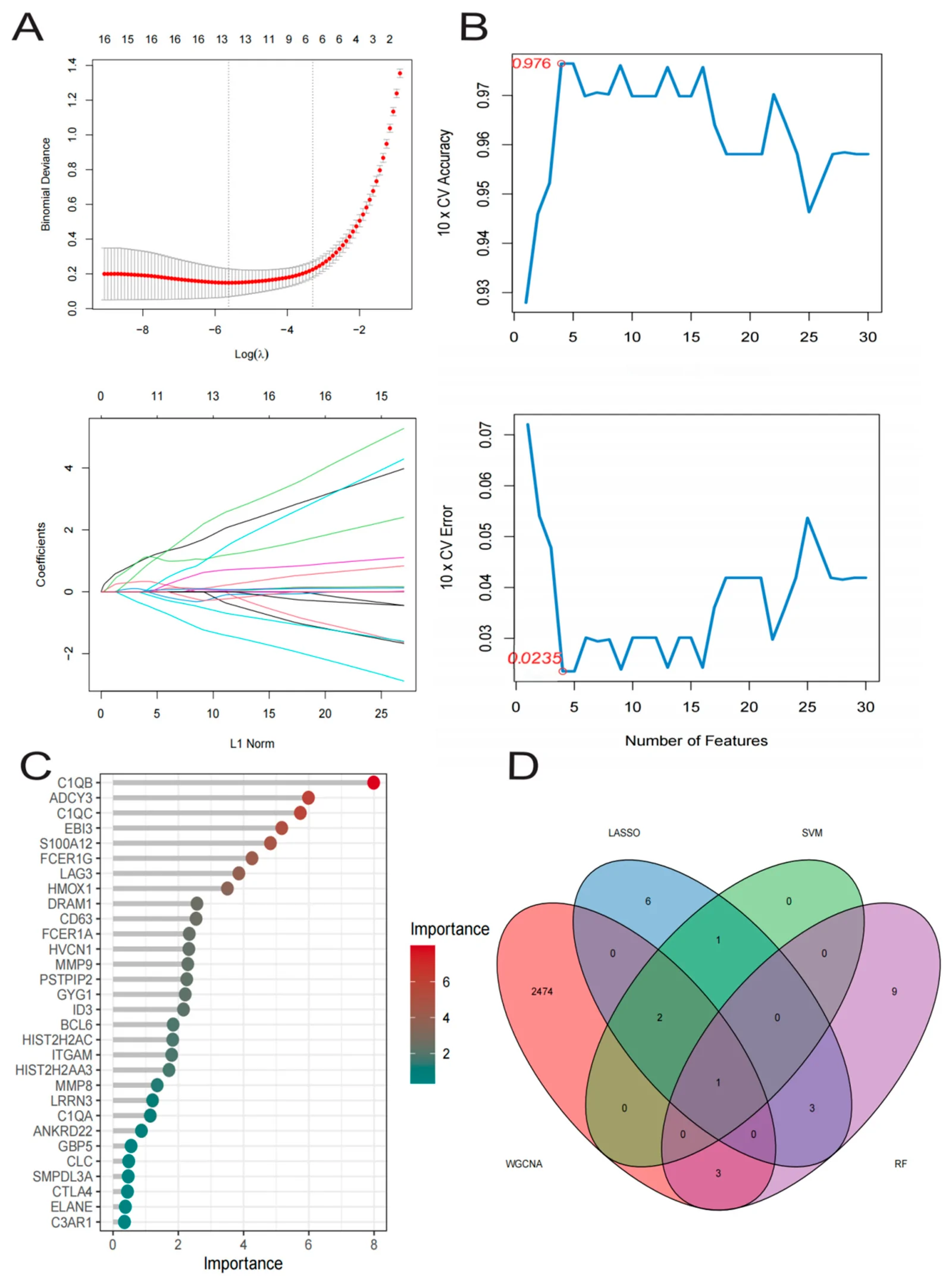
Screening of core genes. (**A**) LASSO analysis of candidate genes. The cross-validation curve shows the deviance as a function of log(λ). The vertical dashed line indicates the optimal λ value, at which 13 genes with non-zero coefficients were retained. The coefficient paths are also shown, in which each colored line represents the coefficient path of one predictor variable. (**B**) SVM feature selection results. The classification performance of different gene subsets is shown. 4 genes were selected at the optimal point as the most discriminative features. (**C**) Random forest (RF) analysis. Genes are ranked by their importance scores, and the top 16 genes were retained for further analysis. (**D**) Venn diagram showing the overlap of genes among WGCNA, LASSO, SVM, and RF.

The overall analysis workflow is summarized in Figure 1. To identify DEGs in malaria, we merged peripheral blood transcriptome data from malaria patients across different regions. PCA showed homogeneous clustering of the integrated samples after batch-effect correction (Figure S1A). We identified 70 DEGs in total, including 62 up-regulated and 8 down-regulated genes (Figure 2A). A heatmap illustrated distinct expression profiles between patients and controls (Figure 2B). Enrichment analysis indicated that the DEGs were primarily involved in immune responses and complement and coagulation cascades (Figure 2C,D). Using WGCNA, we selected a soft-threshold power of β = 13 to approximate a scale-free topology (Figure S1C). Hierarchical clustering yielded multiple co-expression modules (Figure S1B). Correlation analysis showed that the gray module, containing 2480 genes, was the most strongly associated with malaria (Figure 2E). To identify hub genes, we applied LASSO, SVM and RF for further hub variable identification. LASSO, SVM, and RF methods respectively identified 13, 4, and 16 genes in the training set (Figure 3A–C). The intersection of WGCNA and the three machine learning methods yielded a single common gene: C1QB (Figure 3D).

**Figure 4:**
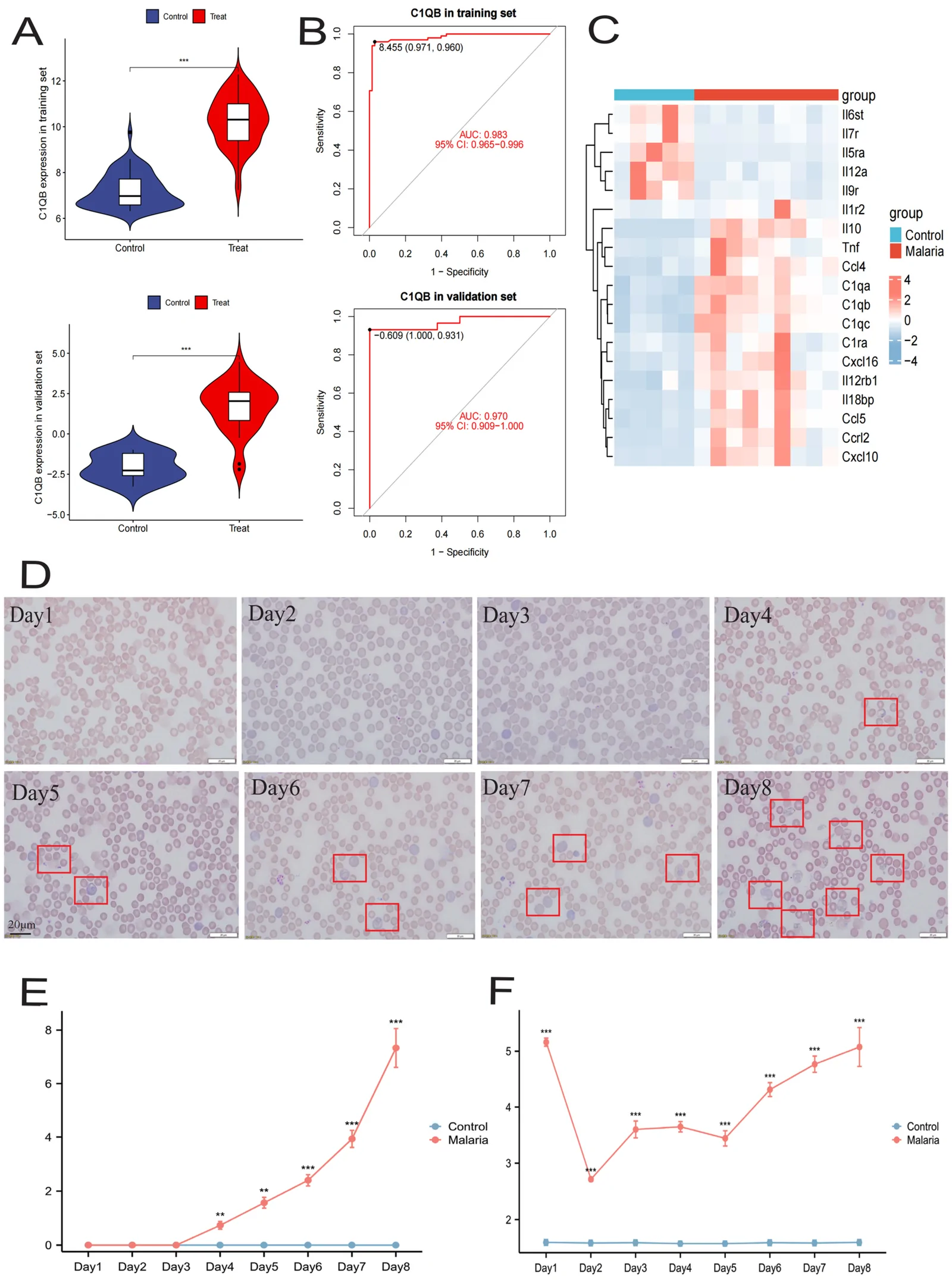
C1QB expression and ROC analysis. (**A**) C1QB levels in the training and validation sets. (**B**) ROC curves of C1QB in both sets. (**C**) Heatmap of C1QB and associated inflammatory genes in malaria and control mice. (**D**) Peripheral blood smears (Giemsa stain) from day 1 to day 8 post-infection. Red boxes highlight the infected red blood cells. (**E**) Parasitemia dynamics (days 1–8). (**F**) Temporal change in peripheral blood C1QB measured by ELISA (days 1–8). Data are mean ± SEM. Two-way repeated-measures ANOVA with Tukey’s test: significant treatment × time interaction. Post hoc comparisons: ** *p* < 0.01, *** *p* < 0.001 for model vs. control (Tukey-adjusted).

A significant pattern of upregulation of C1QB in treat group (malaria) was observed in the training and validation sets (*p*  <  0.001) (Figure 4A). To evaluate the diagnostic ability of C1QB, ROC curve analysis was performed in both sets. The results showed that the AUC value for C1QB in the training set was 0.983 (95% CI: 0.965–0.996) (Figure 4B), and the AUC value for C1QB in the validation set was 0.970 (95% CI: 0.909–1.000), indicating high sensitivity and specificity to distinguish between malaria and control. Therefore, C1QB was identified as a central host gene involved in malaria and can serve as a potential diagnostic biomarker.

**Figure 5:**
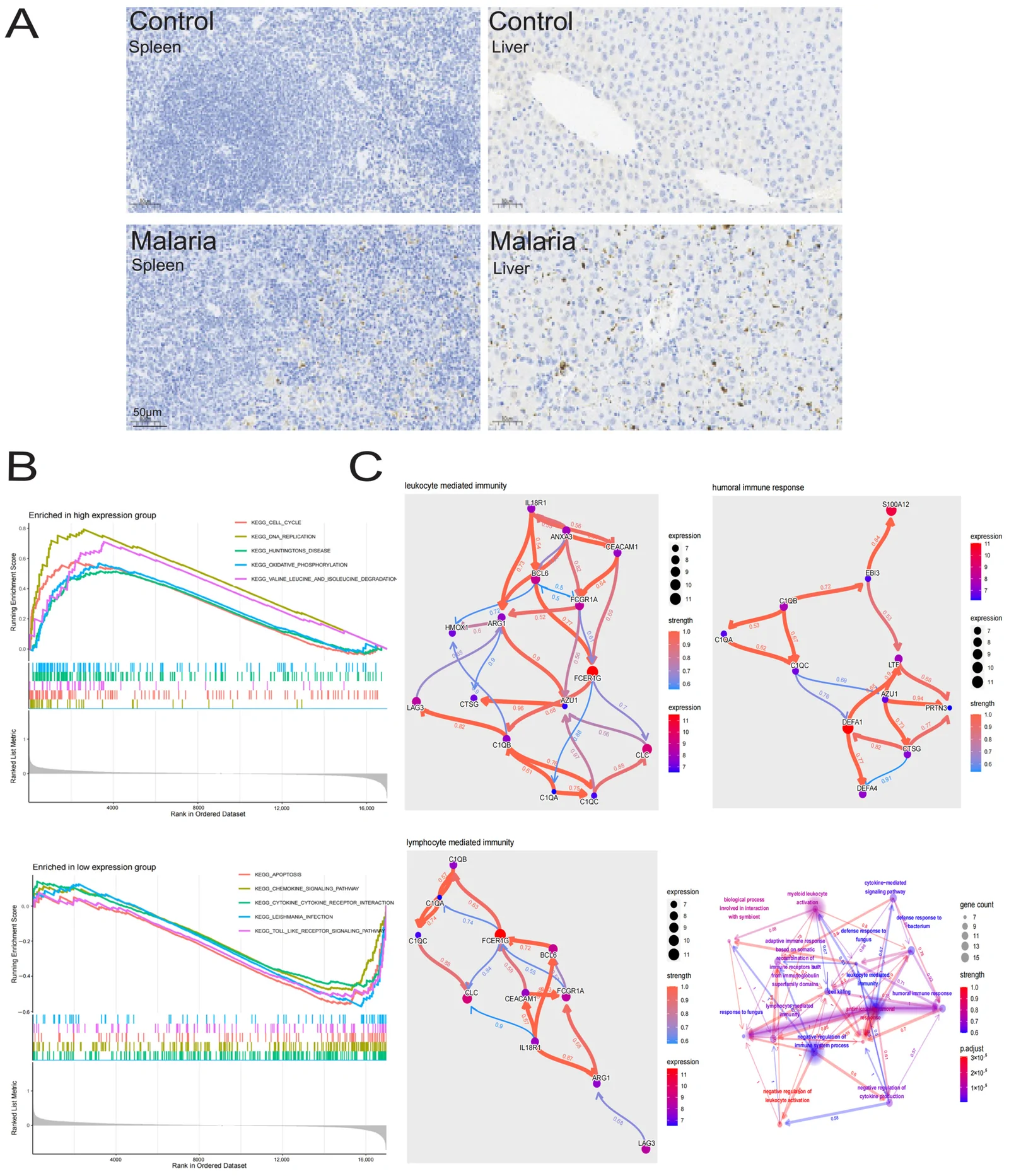
Immune features associated with C1QB in malaria. (**A**) C1QB expression in liver and spleen at late infection (day 8, IHC). (**B**) Single-gene GSEA of C1QB. (**C**) Bayesian regulatory network of C1QB.

Analysis of the peripheral blood transcriptome from malaria-infected mice revealed 2428 upregulated genes (Figure S1D), and a heatmap highlighted changes in inflammation-related molecules, including C1QB, C1QA, and C1QC (Figure 4C). Giemsa staining of blood smears revealed that during the early stage (days 0–3) of *Plasmodium berghei* ANKA (*Pb*ANKA) infection, no parasites were detectable. By day 4, a few infected red blood cells were visible under light microscopy, while by day 8, many were observed (Figure 4D,E). In contrast, C1QB expression increased markedly (*p* < 0.001) as early as the initial phase of infection—even before infected red blood cells became visible on Giemsa-stained smears. C1QB levels rose sharply on day 1 post-infection, moderated slightly on day 2 (still higher than in the normal group), and then climbed significantly again from day 5 onward as the infection progressed (*p* < 0.001, Figure 4F). At the same time, given that the liver and spleen are major target organs in malaria, IHC showed elevated C1QB expression in the liver and red pulp of spleen (Figure 5A).

### 2.2. C1QB Is Associated with Immune Damage in Malaria

**Figure 6:**
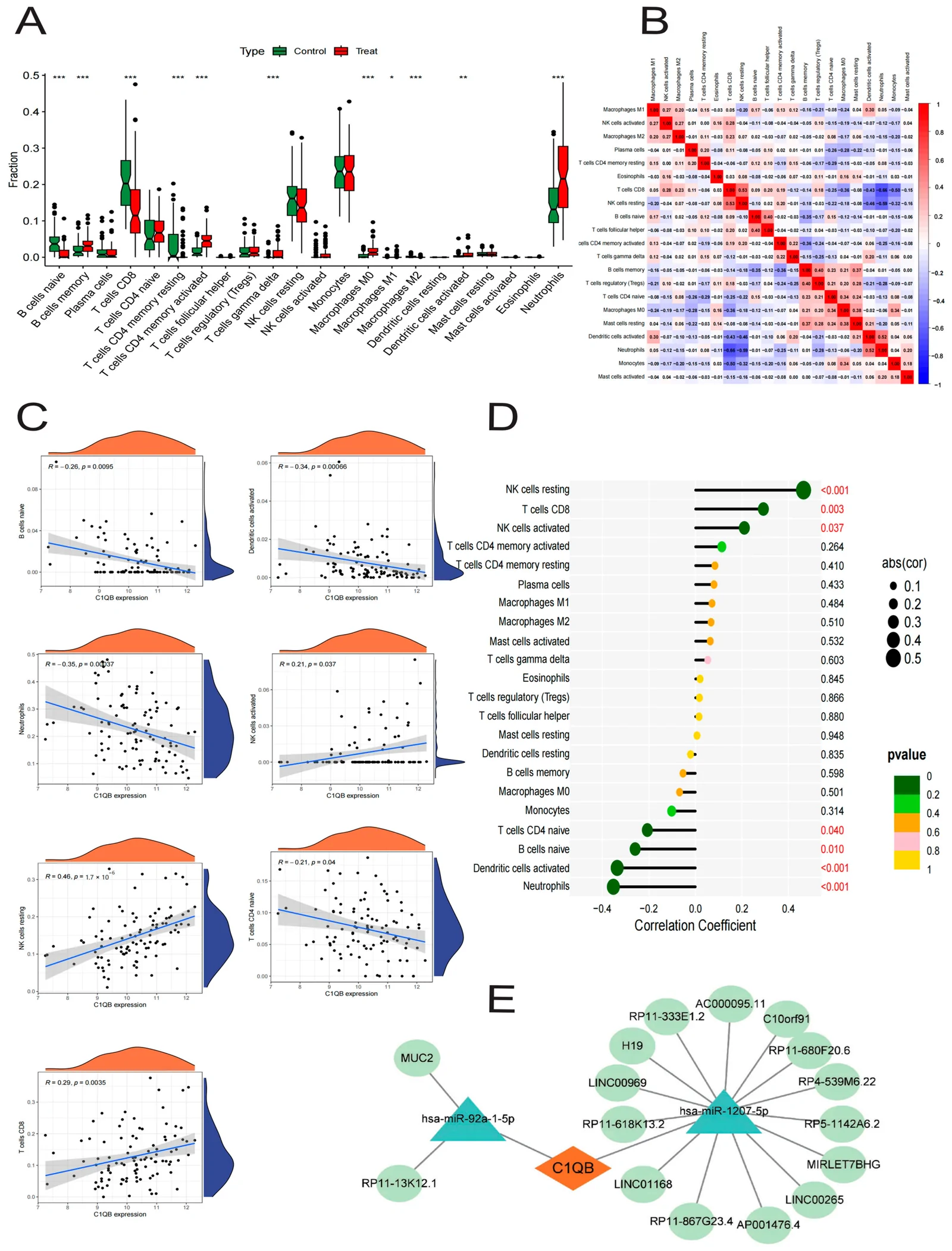
Immune infiltration and ceRNA regulatory network of C1QB. (**A**) Immune-cell infiltration profile (22 cell types) in the training set. Box plots represent the distribution of immune cell infiltration levels. * *p* < 0.05, ** *p* < 0.01, *** *p* < 0.001. (**B**) Correlation among key immune cells in the training set. (**C**,**D**) Correlation between C1QB and immune-infiltrating cells. (**E**) ceRNA network of C1QB.

GSEA was conducted to explore the biological processes linked to the feature gene. Apoptosis, chemokine signaling pathway, cytokine–cytokine receptor interaction, leishmania infection and toll-like receptor signaling pathway were significantly involved in C1QB-low samples. And cell cycle, DNA replication, Huntington’s disease, oxidative phosphorylation, valine, leucine and isoleucine degradation were significantly involved in C1QB-high samples (Figure 5B). Bayesian regulatory network analysis revealed complex pathway regulation in malaria, with C1QB involved in multiple pathways. Specifically, C1QB regulates LAG3, HMOX1, and C1QC in leukocyte-mediated immunity, EBI3, C1QA, and C1QC in humoral immune response, and C1QA and C1QC (while being regulated by FCER1G) in lymphocyte-mediated immunity. The pathway diagram further shows that these three pathways are closely linked to the cell killing pathway (Figure 5C). Immune infiltration analysis revealed a significant increase in activated dendritic cells and neutrophils (Figure 6A), which were negatively correlated with C1QB expression (Figure 6C). Conversely, naive B cells and CD8 T cells were markedly decreased (Figure 6A). Naive B cells showed a negative correlation with C1QB, whereas CD8 T cells showed a positive correlation (Figure 6C). Correlation analysis further indicated that activated dendritic cells were strongly associated with neutrophils, and both were highly negatively correlated with CD8 T cells (Figure 6B). These results indicate that these four cell types play crucial and multifaceted roles in C1QB-regulated immune pathways and are potentially linked to immune damage in malaria. To investigate post-transcriptional regulation of the identified gene, we constructed a competing endogenous RNA (ceRNA) network. This network contained 18 confirmed interactions, including C1QB, 2 miRNAs, and 16 lncRNAs (Figure 6E).

**Figure 7:**
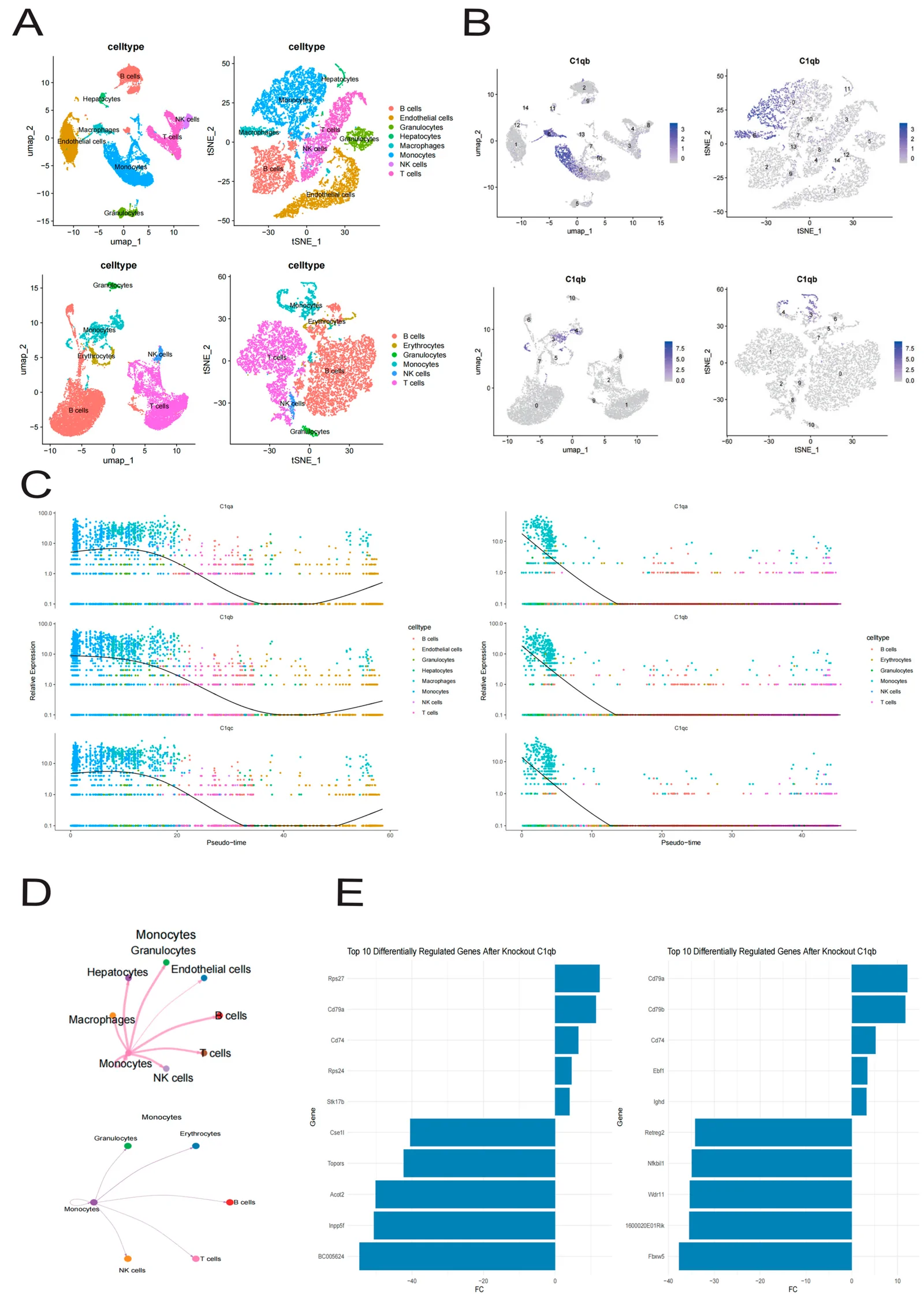
Single-cell analysis of C1QB expression in liver and spleen of *Pb*ANKA-infected mice. (**A**) Uniform Manifold Approximation and Projection (UMAP) and t-distributed Stochastic Neighbor Embedding (t-SNE) plots displaying 8 annotated cell types in the liver (**upper panel**) and 6 cell types in the spleen (**lower panel**). Cell types were identified based on canonical marker genes. (**B**) Feature plots showing C1QB expression levels across different cell types in the liver (**upper**) and spleen (**lower**). Color intensity indicates relative expression (log-normalized counts). (**C**) Pseudotime trajectory analysis of C1QB, C1QA, and C1QC expression. Cells are ordered along a differentiation trajectory (pseudotime) using Monocle 2. Expression levels of C1QB, C1QA, and C1QC are shown as a function of pseudotime, with different colors representing distinct cell types. (**D**) Cell–cell communication network inferred by CellChat. Nodes represent cell types. Edge thickness indicates interaction strength. The network highlights communication patterns involving monocytes in both liver and spleen. (**E**) Virtual knockout (scTenifoldKnk) analysis of C1QB. The plot shows the top differentially regulated genes (e.g., Rps27, Cd79a, Cd79b, Cd74) upon in silico deletion of C1QB.

To further explore the temporal and spatial mechanisms of C1QB in malaria, we performed single-cell RNA sequencing. Quality control confirmed stable sequencing depth, gene number, and mitochondrial content across samples (Liver: Figure S1E,F. Spleen: Figure S2A,B). Bubble chart visualization of marker genes enabled accurate cell-type annotation, identifying 14 major clusters in the liver and 10 in the spleen (liver: Figure S2C,E. Spleen: Figure S2D,F). UMAP and t-SNE plots revealed 8 distinct cell types in the liver and 6 in the spleen (Figure 7A). Notably, monocytes were the most abundant in the liver, while T and B cells predominated in the spleen, suggesting persistent and significant immune dysregulation in malaria. C1QB was mainly expressed in mononuclear cells of both organs and implicated in immune regulation (Figure 7B). Pseudotime trajectory analysis indicated that C1QB dynamically regulates immunity, with expression peaking in early developmental monocytes and declining later. C1QA and C1QC followed the same trend (Figure 7C). Further analysis revealed extensive cell–cell communication involving monocytes in both the liver and spleen (Figure 7D).

### 2.3. Blocking C1q Alleviates Immunopathology in Malaria

We applied scTenifoldKnk to investigate gene expression and immune changes after virtual knockout of C1QB in malaria. Unlike capturing large-scale transcriptional shifts, this method identifies critical network nodes truly regulated by C1QB. The most affected genes—Rps27, Cd79a, Cd79b, and Cd74—are associated with B cell development, activation, and antigen presentation, indicating enrichment in B cell-related pathways (Figure 7E). These findings suggest that C1QB plays a key role in adaptive immunity during malaria. Previous studies have shown that C1QB does not act alone. Heatmap analyses in humans and mice (Figure 2B and Figure 4C) and single-cell trajectory analysis (Figure 7C) revealed that C1QB, C1QA, and C1QC are highly correlated. Together, they encode the six subunits (18 polypeptide chains) of C1q, which assemble into the functional C1q complex. Elevated C1q expression may contribute to malaria-associated inflammation and tissue damage. To test this, we used an antibody that binds specifically to C1q and blocks its downstream activity.

**Figure 8:**
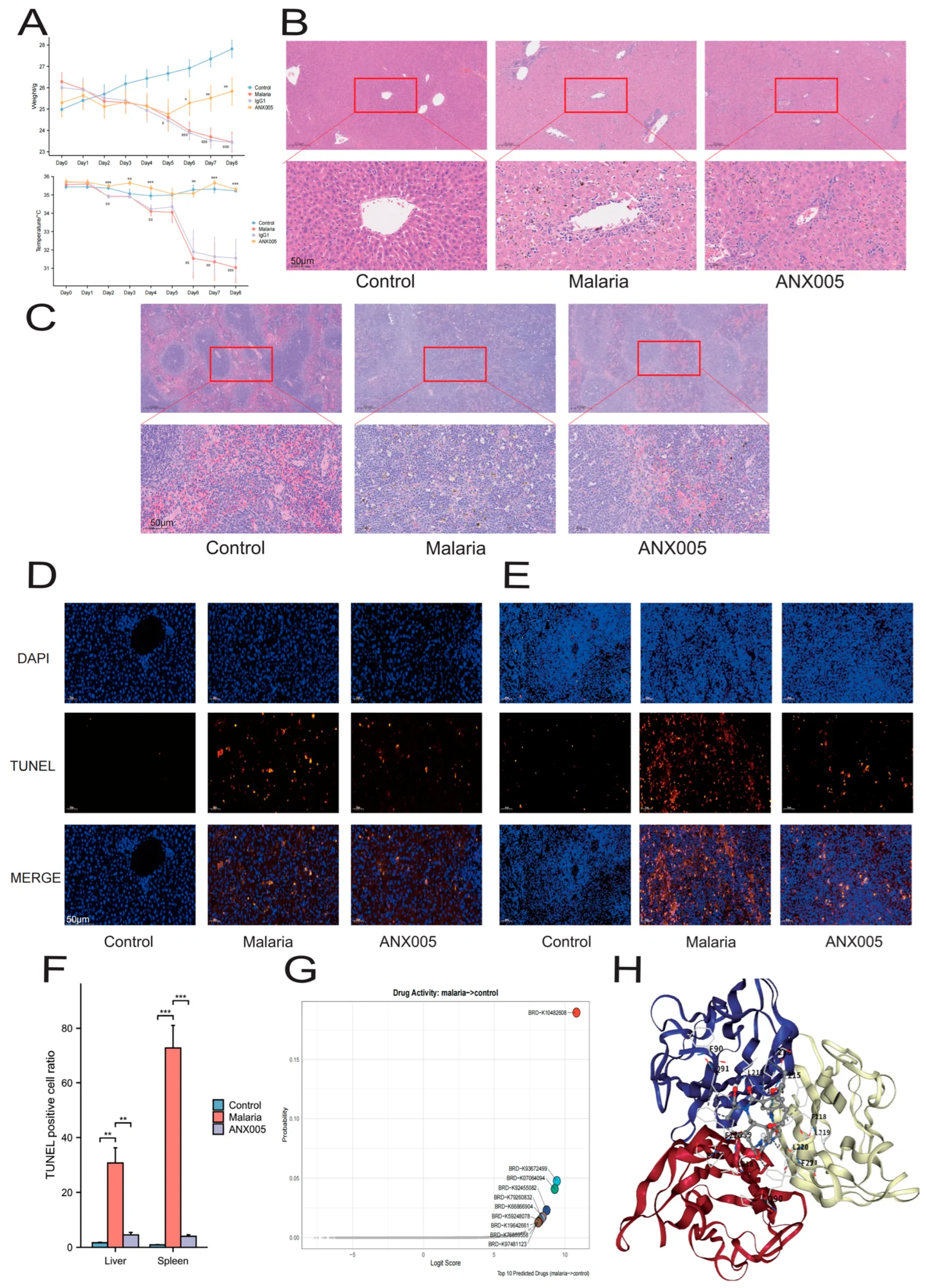
C1q-targeted therapy in *Pb*ANKA-infected ICR mice. (**A**) Body weight (g) and body temperature (°C) over 8 days. Mice were infected with *Pb*ANKA and treated with anti-C1q antibody (ANX005, 20 mg/kg i.p.) or IgG1 isotype control (BE0083) on days 0, 3, and 6. Normal mice served as uninfected controls (*n* = 6/group). Data are mean ± SEM. Two-way repeated-measures ANOVA with Tukey’s test: significant treatment × time interaction for both weight and temperature. Post hoc comparisons: * *p* < 0.05, ** *p* < 0.01, *** *p* < 0.001 for ANX005 vs. malaria; # *p* < 0.05, ## *p* < 0.01, ### *p* < 0.001 for control vs. malaria (Tukey-adjusted). (**B**,**C**) H&E staining of liver (**B**) and spleen (**C**). Scale bar = 50 μm. (**D**,**E**) TUNEL staining of liver (**D**) and spleen (**E**). Orange: TUNEL-positive cells. Blue: DAPI. Scale bar = 50 μm. (**F**) Quantification of TUNEL-positive cells (% of total). Data are mean ± SEM. Two-tailed Student’s *t*-test: ** *p* < 0.01, *** *p* < 0.001 vs. malaria. (**G**) Top candidate drugs predicted by DrugRefLector. (**H**) Molecular docking of BRD-K10482608 with C1q complex (C1qb/C1qa/C1qc, PDB ID: 1pk6). Lowest-energy pose (−7.8 kcal/mol) with key residues labeled.

The results showed that, while normal mice steadily gained weight, mice in the malaria-infected and IgG1 control groups lost weight continuously. In contrast, mice treated with C1q antibody began regaining weight at the later stage (days 5–8). Body temperature remained stable in normal mice but dropped markedly in the malaria and IgG1 groups (days 5–8). Mice receiving C1q antibody maintained a largely stable body temperature, with significant differences between the ANX005 and malaria groups indicated by asterisks (* *p* < 0.05, ** *p* < 0.01, *** *p* < 0.001). Significant differences between the control and malaria groups were indicated by hash marks (# *p* < 0.05, ## *p* < 0.01, ### *p* < 0.001), as detailed in Figure 8A.

H&E staining of the liver showed well-structured tissue, clear hepatocyte arrangement, and no inflammatory infiltration in normal mice. In infected mice, the sinusoidal structure was disrupted with extensive inflammatory infiltration, widespread hepatocyte ballooning, and fatty degeneration. The antibody-treated group showed markedly reduced degeneration and infiltration (Figure 8B). In the spleen, the boundary between white and red pulp was clear under normal conditions. In infected mice, this boundary became blurred, with white pulp atrophy and structural disorganization, indicating immune imbalance. Red pulp showed massive immune cell infiltration, scattered necrotic foci, and apoptotic cells, along with abundant brown-yellow hemosiderin deposits (markers of red blood cell destruction). In the treated group, the white-red pulp boundary was relatively clear, infiltration and apoptosis were significantly reduced, and hemosiderin deposition decreased (Figure 8C). TUNEL assays confirmed that C1q antibody treatment significantly reduced apoptosis in both liver and spleen cells (Figure 8D–F). These results indicate that C1q promotes inflammation and tissue damage in malaria.

### 2.4. Identification of Potential Therapeutic Drugs

To explore potential therapeutics targeting C1q (C1qb/C1qa/C1qc), we employed the AI framework DrugRefLector on the integrated bulk profile (training set). In total, 10 candidate agents were identified as capable of reversing the malaria signature, with BRD-K10482608 emerging as the most promising (Figure 8G). Subsequent molecular docking showed that BRD-K10482608 binds to C1q with strong affinity (−7.8 kcal/mol), supporting C1q as a valid target (Figure 8H). Together, these results highlight a pathogenic role for C1q in malaria and suggest BRD-K10482608 as a potential therapeutic agent.

## 3. Discussion

### 3.1. C1QB as a Stable Host Biomarker with High Diagnostic Value

Early diagnosis and treatment are critical in malaria, yet a major bottleneck persists: the lack of reliable, host-derived biomarkers for clinical translation. Current diagnostic methods each have significant limitations. Conventional microscopy, while the historical gold standard, is operator-dependent and lacks sensitivity in low-parasite-density infections [10]. RDTs, which detect antigens like HRP2 or pLDH, offer a practical alternative but exhibit variable performance due to factors such as parasite density, species, and antigen expression levels [10,15,16]. Molecular techniques (PCR, LAMP) provide high sensitivity and specificity, particularly for low parasitemia, but their reliance on specialized equipment and expertise restricts use in resource-limited settings [10,17]. This landscape underscores the clear need for novel diagnostic biomarkers.

Our study employed a multi-cohort integrative analysis, combining transcriptomic datasets from diverse geographical regions (East Africa, West Africa, and Southeast Asia). This strategy is essential to mitigate regional biases arising from variations in parasite strains, host genetics, and environmental factors, thereby enhancing the generalizability and robustness of identified biomarkers. We implemented a dual computational framework for rigorous biomarker screening. First, WGCNA was used to identify gene modules strongly correlated with malaria infection status, capturing coordinated immune responses beyond single-gene analysis. Subsequently, multiple machine learning algorithms were applied for feature selection and model construction. Key hyperparameters were optimized via grid search or Bayesian optimization to ensure peak model performance. Validating findings across these distinct algorithms, each with different underlying assumptions, significantly strengthens the robustness of our results and minimizes risks of overfitting or algorithm-specific artifacts.

Focusing on host immune biomarkers is particularly advantageous. Unlike direct pathogen detection, which can be compromised by antigenic variation or low parasitemia, host response signatures provide a stable and integrative readout of the disease state [18]. The high AUC values achieved by our final biomarker panel underscore its strong diagnostic potential. An AUC approaching 1.0 indicates an excellent ability to discriminate malaria patients from controls, suggesting these markers could form the basis for developing highly accurate auxiliary diagnostic tools.

### 3.2. C1QB Promotes Immune Dysregulation in Malaria

C1QB is a key structural component of the C1q complex, which initiates the classical complement pathway. The functional C1q complex is a hexamer with a bouquet-like structure, assembled from polypeptide chains encoded by three distinct genes: C1QA, C1QB, and C1QC [19]. Under physiological conditions, C1QB expression is low, but it is significantly upregulated in various disease states [20,21,22]. This expression is tightly regulated: transcription factors such as PU.1 and IRF8, which respond to inflammatory signals, modulate C1QB transcription during immune activation [23]. Furthermore, epigenetic mechanisms, including DNA methylation and histone modifications at the C1QB locus, fine-tune its expression in a cell type- and context-dependent manner [24]. Notably, C1QB has been identified as a diagnostic biomarker and therapeutic target in other immune-related diseases, such as systemic lupus erythematosus and neurological disorders [7,25]. Therefore, C1QB represents a promising candidate as a sensitive diagnostic biomarker for malaria, and its inhibition could be a viable therapeutic strategy, a hypothesis strongly supported by the findings of our present study.

GSEA revealed distinct biological states associated with C1QB expression. Samples with low C1QB were enriched for pathways characteristic of an active innate immune response, where apoptosis may reflect the immune-mediated clearance of infected cells or dysregulated cell death in severe malaria. Conversely, samples with high C1QB showed enrichment in pathways suggesting a shift toward cellular homeostasis and dampened inflammation, possibly indicative of effective early-stage parasite control or recovery. This implicates C1QB in promoting innate immunity and *Plasmodium* clearance, highlighting its protective role in malaria.

Bayesian regulatory network analysis further demonstrated that C1QB participates in multiple interconnected immune processes. These processes converge on the cell-killing pathway, suggesting C1QB helps coordinate both antibody-dependent and cell-mediated cytotoxic mechanisms against the parasite. This positions C1QB not merely as a passive biomarker but as an active orchestrator of the balance between protective immunity and immunopathology.

Immune infiltration findings support this view. The observed increase in neutrophils and activated dendritic cells aligns with typical acute-phase responses in active malaria [26], while the reduction in naive B cells likely reflects their activation and differentiation into antibody-secreting cells [27]. The positive correlation between C1QB and CD8^+^ T cells further suggests that higher C1QB expression may support adaptive cellular immunity.

Collectively, these results underscore the central role of the complement system, where C1q—with C1QB as a key subunit—initiates the classical pathway. This pathway critically bridges innate and adaptive immunity by opsonizing pathogens, promoting phagocytosis, and modulating immune cell responses. In malaria, complement activation thus presents a double-edged sword, capable of controlling parasitemia while also contributing to inflammation-driven pathology, making C1QB a functionally relevant hub. Finally, while we identify C1QB as a robust host biomarker, its potential regulation within a ceRNA network warrants mention. Shared miRNA response elements suggest complex post-transcriptional control that could influence both its diagnostic utility and regulatory function in malaria, offering a direction for deeper mechanistic exploration.

### 3.3. Spatiotemporal C1QB Dynamics in Monocytes Drive Early Host Defense

Our findings reveal a critical, time-sensitive role for C1QB in the early host response to *Plasmodium* infection. Notably, C1QB expression was significantly elevated as early as day 1 post-infection, before parasites were detectable by blood smear microscopy. This temporal dissociation positions C1QB not merely as a correlate of parasite burden, but as a rapid, host-derived sentinel of infection. It suggests that classical complement activation is triggered very early—potentially by immune complexes or danger signals released during the hepatic stage—offering a diagnostic window earlier than current parasitological methods.

Single-cell RNA sequencing provided cellular resolution to this early signal. Monocytes localized in the liver and spleen emerged as the primary hubs of C1QB expression during malaria. This finding is highly significant: given that the C1q complex is predominantly produced by mononuclear phagocytes, it pinpoints the infected liver and spleen as major anatomical sites where complement cascade initiation occurs. This spatial specificity aligns with organ-specific malaria pathogenesis and indicates that hepatic and splenic monocytes are among the first responders, deploying C1q to opsonize sporozoites or merozoites and orchestrate downstream immune responses.

Pseudotime trajectory analysis further revealed a dynamic regulatory program. C1QB expression peaked in early developmental monocytes and subsequently declined, a trend mirrored by its partners C1QA and C1QC. This pattern indicates that C1q production is tightly regulated during monocyte differentiation—a transient burst of synthesis is crucial for launching initial immune defense, followed by downregulation to prevent excessive inflammation or immunopathology. This aligns with the findings from animal experiments.

Finally, cell–cell communication analysis showed that monocytes in the liver and spleen engage extensively with diverse cell types, including hepatocytes, endothelial cells, and other immune subsets. This widespread crosstalk suggests that the early C1q signal is rapidly broadcast, mediating enhanced clearance of infected cells, modulation of endothelial activation, and recruitment or instruction of adaptive immune cells [28,29]. Collectively, these data depict a coherent picture: C1QB marks a pivotal early event in which tissue-resident monocytes, upon sensing malaria infection, dynamically upregulate and release C1q to coordinate a localized yet systemic immune response, bridging immediate pathogen control with adaptive immunity.

### 3.4. C1q Contributes to Immunopathology

Our in vivo intervention study provides direct functional evidence that blocking C1q activity confers significant protection against severe malaria pathology. Mice treated with a C1q-neutralizing antibody showed marked improvements in key clinical and pathological outcomes: maintenance of body temperature and prevention of weight loss indicate a reduction in systemic disease burden, while the marked decrease in liver and spleen injury points to a direct protective effect at the primary sites of parasite replication and immune activation. The reduction in hepatosplenic apoptosis is especially critical, as it suggests that C1q inhibition curbs excessive immune-mediated cell death, a key contributor to organ dysfunction in malaria [30,31]. Collectively, these results underscore that pathological complement activation via the classical pathway is a major driver of disease severity, and its blockade shifts the host response from harmful inflammation toward more controlled immunity.

This finding decisively transforms C1QB from a correlative biomarker into a functional disease mediator. Our data confirm that C1QB is not merely a passive indicator of infection but an active participant in the immunopathological cascade. The therapeutic efficacy of C1q blockade validates the predictions from our prior multi-omics analyses—namely, that C1q sits at a critical hub regulating immune effector mechanisms such as cell killing and inflammation. By inhibiting this hub, we uncouple the beneficial, parasite-clearing functions of the immune response from its harmful, tissue-destructive consequences. Thus, C1QB represents a dual-function target: its expression level serves as a robust diagnostic and prognostic biomarker, while its protein product (as part of the C1q complex) offers a tractable therapeutic node for immunomodulation. This dual role highlights the translational value of host-response signatures, providing a strategy for developing adjunctive therapies aimed at modulating the immune response to improve outcomes in severe malaria.

### 3.5. Clinical Translational Significance

Our study identifies C1QB as a pivotal molecule with dual translational potential in malaria. Its early upregulation prior to detectable parasitemia offers a critical diagnostic window for pre-symptomatic intervention. Furthermore, in vivo C1q blockade alleviated disease pathology, establishing C1q/C1QB as a functional mediator of immunopathology and a viable target for immunomodulatory therapy in severe malaria. Supporting this therapeutic direction, our computational drug repurposing analysis via DrugRefLector identified BRD-K10482608 as a candidate compound with predicted efficacy against malaria. Notably, subsequent molecular docking simulations demonstrated a strong binding affinity between BRD-K10482608 and the C1q protein complex. This prediction not only reinforces the druggability of the C1q pathway but also provides a specific, testable lead compound. Future studies validating the efficacy of BRD-K10482608 in experimental models could directly translate our mechanistic insights on C1QB into a novel host-directed therapeutic strategy.

### 3.6. Limitations

This study elucidates the role of C1q/C1QB in malaria immunopathology and diagnosis, while also acknowledging several limitations that inform future research directions. First, although we integrated peripheral blood transcriptome data from multiple regions, corrected for batch effects, and independently validated C1QB’s diagnostic performance, residual confounding from genetic background, infection stage, or comorbidities may persist. Furthermore, the human transcriptomic datasets used were predominantly derived from patients infected with *P. falciparum*. Given that immunopathological mechanisms and therapeutic responses differ among *Plasmodium* species, the generalizability of our findings to infections caused by *Plasmodium vivax* or *Plasmodium ovale* requires further validation in multi-species cohorts. Therefore, confirming C1QB’s robustness and clinical utility in larger, clinically well-characterized, and species-diverse cohorts remains essential for clinical translation.

Second, while we demonstrated early elevation and high expression of C1QB in peripheral blood and key target organs—the liver and spleen—malaria is a systemic infection that can involve the brain and kidneys in severe disease. Future work should examine C1QB expression and its pathological relevance in other affected tissues, such as the brain microvascular endothelium and renal glomeruli, to better evaluate its tissue specificity and safety as a therapeutic target.

Finally, molecular docking indicates that the virtually screened compound BRD-K10482608 binds efficiently to C1q, supporting its potential as a targeted therapy. Assessing the efficacy and safety of BRD-K10482608 in preclinical models and advancing it toward clinical studies are necessary steps to translate these findings into therapeutic applications.

Despite these limitations, our study provides novel insights into the immunopathological mechanisms of *P. falciparum* malaria. Future work should focus on advancing the clinical translation of C1q-targeted strategies, extending these findings to other *Plasmodium* species, and further elucidating its precise regulatory roles in severe malaria, thereby paving the way for precision diagnosis and treatment of the disease.

## 4. Materials and Methods

### 4.1. Study Design

We employed an integrative approach, combining transcriptomic data from GEO with WGCNA and three machine-learning algorithms—LASSO, SVM, and RF—to identify central hub genes. The lead candidate, C1QB, was further characterized through GSEA, immune-cell infiltration profiling, construction of Bayesian and ceRNA networks, single-cell RNA sequencing, and perturbation analysis using scTenifoldKnk. Functional validation was performed in a *Pb*ANKA murine model, and intervention assays with an anti-C1q antibody. Finally, AI-based drug prediction coupled with molecular docking was used to evaluate the therapeutic potential of targeting the C1q pathway.

### 4.2. Data Collection and Identification of DEGs in Malaria

We obtained four whole-blood gene expression datasets (GSE117613, GSE35858, GSE34404, GSE119152) [32,33] from GEO (https://www.ncbi.nlm.nih.gov/geo/ (accessed on 1 April 2025)), generated using the Illumina Human HT-12 V4.0 platform and comprising *Plasmodium falciparum* (*P. falciparum*) malaria patients and healthy controls. Data were processed with the Bioconductor package limma (v3.56.0) [34] for background correction, normalization, and probe annotation. Batch effects were assessed by PCA. Three datasets (GSE117613, GSE35858, GSE34404) were merged as a training set, with GSE119152 retained for validation. Differentially expressed genes (DEGs) were identified using |log2FC| > 1 and *p* < 0.05, and visualized via heatmaps (pheatmap) and volcano plots (ggplot2).

### 4.3. Functional Enrichment Analysis

We performed functional annotation and pathway enrichment analysis of the DEGs using the R packages ClusterProfiler (v4.4.0) and DOSE (v3.20.0). Gene Ontology (GO) and KEGG pathway analyses were performed with a significance threshold of q < 0.05 to identify enriched biological processes, molecular pathways, and disease associations [35,36].

### 4.4. WGCNA of DEGs

We performed WGCNA on DEGs using the R package WGCNA (v1.73) [37] to identify gene modules significantly associated with malaria. A scale-free network was constructed by selecting an appropriate soft-thresholding power (β). The gene expression matrix was then transformed into an adjacency matrix, and modules were identified via topological overlap clustering. Highly correlated modules were merged based on module eigengenes (MEs) and visualized in a hierarchical clustering dendrogram. This analysis evaluated the relationships between modules and clinical traits, enabling the selection of host genes associated with malaria for further study.

### 4.5. Screening the Feature Genes by Machine Learning

We employed three machine learning algorithms to screen for robust feature genes. LASSO regression was performed using the glmnet R package (v4.1-4), with the regularization parameter tuned via 10-fold cross-validation [38]. Support Vector Machine–Recursive Feature Elimination (SVM-RFE) was implemented with the e1071 (v1.7-12) and caret packages (v6.0-92), utilizing a radial basis function kernel and 10-fold cross-validation [39]. Additionally, the Random Forest (RF) algorithm, executed via the randomForest package (v4.7-1.1), was applied to rank gene importance; this ensemble method is effective for high-dimensional data and reduces overfitting [40]. Finally, the intersection of genes identified by WGCNA and these three machine learning approaches was taken to define a key host gene associated with malaria.

### 4.6. The Diagnostic Capability and Expression Levels of the Feature Gene

We evaluated the diagnostic potential of the hub gene by performing ROC analysis using the R package pROC (v1.18.5) [41]. Genes with an AUC > 0.7 across all datasets were considered diagnostically relevant. Expression levels of the feature gene were then compared between groups using the limma package.

### 4.7. GSEA and Bayesian Network for the Feature Gene

We performed Gene Set Enrichment Analysis (GSEA) using the clusterProfiler package (v4.8.1) to identify biological pathways linked to the feature gene C1QB. Samples in the training set were stratified into high- and low-expression groups based on median expression, and genes were ranked by log2FC. GSEA was then conducted using this ranked list against the KEGG gene set (c2.cp.kegg.Hs.symbols.gmt), with a significance threshold of adjusted (*p*) < 0.05 and |NES| > 1. Additionally, a Bayesian regulatory network for C1QB was constructed with the CBNplot package based on the GO database [42].

### 4.8. ceRNA Network Construction

To investigate potential post-transcriptional regulation of C1QB in malaria, we constructed a competing endogenous RNA (ceRNA) network comprising lncRNAs, miRNAs, and mRNA. miRNAs were predicted using TargetScan Human 8.0 (https://www.targetscan.org/vert_80/ (accessed on 1 April 2025)), miRDB (http://mirdb.org/ (accessed on 1 April 2025)), and miRanda (http://www.microrna.org/ (accessed on 1 April 2025)) [43,44,45], while miRNA-lncRNA interactions were identified via SpongeScan (http://spongescan.rc.ufl.edu/ (accessed on 1 April 2025)). The resulting regulatory network was visualized with Cytoscape (v3.10.2).

### 4.9. Immune Cell Infiltration Analysis

We assessed immune cell composition in malaria using CIBERSORT-based deconvolution (https://cibersortx.stanford.edu/ (accessed on 1 April 2025)), which estimates the abundances of 22 immune cell types. Subsequently, we evaluated the correlation between the hub gene and key immune cell types, revealing significant associations in malaria samples compared with controls.

### 4.10. Single-Cell Sequencing Analysis

Single-cell RNA-seq data from mouse liver/spleen tissues (OMIX001839) [30] were analyzed to examine the expression of the feature gene. Data processing and analysis were carried out using the Seurat package [46]. Quality control steps excluded cells with fewer than 200 detected genes or with mitochondrial gene percentages exceeding 20%. After normalization, the top 2500 highly variable genes were selected for clustering via t-SNE and UMAP. Cell types were annotated using the scMayoMap algorithm in R, and target-gene expression levels were evaluated across the annotated populations. Cell–cell communication networks were inferred with the CellChat package. Pseudo-time analysis of target-gene expression within specific cell types was performed using monocle2. Finally, ScTenifoldKnk [47] was employed to identify knockout (KO) effects of the hub gene in the target cell type.

### 4.11. Malaria Animal Model Establishment

Female ICR mice (24–28 g) were obtained from Guangzhou Ruige Biological Technology Co., Ltd. (Guangzhou, China) and housed under a 12 h light/dark cycle at 22–25 °C with food and water available ad libitum. *Pb*ANKA parasites were provided by Guangzhou University of Chinese Medicine. Parasite cryovials were thawed in a 37 °C water bath, and mice were then intraperitoneally inoculated with 5 × 10^6^ parasitized erythrocytes. All animal procedures were approved by the institutional ethics committee (Permit No. PZ25038).

### 4.12. Parasitemia Determination

Blood was collected from the tail of *Pb*ANKA-infected mice, smeared onto microscope slides, and air-dried. Each slide was then stained with Giemsa solution I for 1 min, followed by Giemsa solution II (Yeasen, Shanghai, China) for 5 min at room temperature. Infected erythrocytes were counted under a light microscope with a 100 × oil-immersion objective, and parasitemia was calculated as follows:

$$parasitemia=\frac{number\;of\;parasitized\;erythrocytes}{total\;number\;of\;erythrocytes}\times 100\%$$

### 4.13. Transcriptome Sequencing of Mouse Peripheral Blood

Total RNA was extracted from peripheral blood of mice with 10–20% parasitemia using TRIeasy™ Total RNA Extraction Reagent (Yeasen, Shanghai, China) and sent to HaploX (China) for RNA-seq. Each group included at least five biological replicates, all processed under identical conditions. After RNA purity and integrity were assessed, libraries were sequenced on an Illumina platform.

### 4.14. Serum C1QB Measurement by ELISA

We monitored C1QB expression dynamics by sacrificing mice on each of the 8 consecutive days (*n* = 6 mice per day) and collecting peripheral blood immediately after euthanasia. Blood was drawn into plain tubes, clotted at room temperature for 30 min, and centrifuged at 1500 rpm for 10 min to obtain serum, which was stored for subsequent assays. Serum C1QB levels were measured using a Mouse C1QB ELISA Kit (FineTest, Wuhan, China) according to the manufacturer’s instructions. Briefly, 100 µL of assay buffer, standards, or serum samples was added to designated wells. After incubation and washing, the detection antibody was added, followed by substrate solution. The reaction was stopped with 100 µL of stop solution, and absorbance was read at 450 nm.

### 4.15. C1QB Expression in Liver and Spleen by Immunohistochemistry (IHC)

Mice were sacrificed on day 8 post-infection, and the liver and spleen were immediately harvested. After removing adherent fat and connective tissues, the organs were rinsed with ice-cold PBS and blotted dry. Tissue samples were then fixed in 4% paraformaldehyde (PFA) for 24 h at room temperature, dehydrated through a graded ethanol series, cleared in xylene, and embedded in paraffin. Paraffin-embedded sections (4–5 μm thick) were deparaffinized, rehydrated, and subjected to antigen retrieval in citrate buffer (pH 6.0). Endogenous peroxidase activity was blocked with 3% H_2_O_2_, followed by incubation with 5% BSA to reduce nonspecific binding. Sections were then incubated overnight at 4 °C with an anti-C1QB primary antibody. After washing, an HRP-conjugated secondary antibody was applied, and signals were visualized using DAB substrate. The reaction was stopped, and nuclei were counterstained with hematoxylin. Finally, stained sections were scanned, and representative fields were captured using the KFSlideOS system.

### 4.16. Anti-C1q Antibody Treatment

Since C1QB encodes a subunit of complement C1q, we assessed the therapeutic effect of blocking C1q using an anti-C1q antibody (ANX005, MedChemExpress, Monmouth Junction, NJ, USA). Mice were administered either the anti-C1q antibody (20 mg/kg body weight) or an IgG1 isotype control (BE0083, BioXcell, Lebanon, NH, USA) via intraperitoneal injection (i.p.) [48]. Treatment was initiated 1 h after *Pb*ANKA inoculation, a timing chosen to ensure that complement blockade was established before the peak of early complement activation. This early intervention allowed us to assess the therapeutic impact of C1q blockade on the entire course of infection. The treatment was repeated every 3 days for a total of 8 days, following a regimen previously established in the literature for sustained complement inhibition [7], thereby providing continuous coverage throughout the acute phase of infection.

### 4.17. H&E Staining and TUNEL Assay

Liver and spleen tissues were fixed, paraffin-embedded, and sectioned at 4 μm. After deparaffinization and rehydration, sections were stained with hematoxylin for 5 min and eosin for 3 min and then dehydrated, cleared, and mounted with neutral resin for observation. Apoptosis was assessed using a TUNEL System kit (Promega, Madison, WI, USA) according to the manufacturer’s instructions. Briefly, paraffin sections were pretreated, incubated with equilibration buffer followed by rTdT incubation buffer, and placed in a humidified chamber at room temperature in the dark for 60 min. The reaction was stopped with 2× SSC solution, nuclei were counterstained with DAPI, and slides were mounted with anti-fade medium for imaging.

### 4.18. AI-Driven Drug Prediction and Molecular Docking Identify C1QB as a Potential Therapeutic Target

To evaluate whether C1QB could be a therapeutic target for malaria, we used a recently published active deep-learning framework (DrugRefLector) that predicts candidate drugs based on transcriptomic expression profiles. This framework has demonstrated high predictive efficiency [49]. Molecular docking between the C1q complex and the candidate drug was performed using CB-DOCK2 [50]. The 3D structure of the top-ranked drug was downloaded from PubChem, and the structure of the C1q complex (C1qa/C1qb/C1qc, PDB ID: 1pk6) was retrieved from the RCSB PDB database. A binding energy lower than −7 kcal/mol was considered indicative of strong binding.

### 4.19. Statistical Analysis

Experimental replicates: All experiments were performed in triplicate (*n* = 3 independent biological replicates), unless otherwise stated, to ensure reproducibility of the findings. All statistical analyses were conducted with R software (version 4.2.1). For comparisons between two groups, Student’s unpaired two-tailed *t*-test was used. For comparisons among multiple groups, one-way analysis of variance (ANOVA) followed by Tukey’s post hoc test was applied. When two factors were involved, a two-way ANOVA with Bonferroni correction was used for post hoc comparisons. Spearman’s correlation analysis was used to assess the relationship between gene expression and immune cell infiltration. A *p*-value < 0.05 was considered statistically significant.

## 5. Conclusions

This study systematically presents a multi-omics analysis of the role of C1QB in a murine model of *Plasmodium falciparum* malaria. Our findings suggest that C1QB may serve as a potential diagnostic biomarker for *P. falciparum* malaria, is widely involved in immune regulation, and is associated with liver and spleen tissue damage in the murine model. The C1QB/C1q complex is identified as a potential druggable target in this context. These results are based on the transcriptomic and experimental data from the current study and require further validation in human cohorts and across other *Plasmodium* species.

## Data Availability

The datasets used and/or analyzed during the current study are available from the corresponding author on reasonable request.

## Supplementary Materials

The following supporting information can be downloaded at: https://www.mdpi.com/article/10.3390/ijms27167459/s1.

## Abbreviations

The following abbreviations are used in this manuscript:

WGCNA	Weighted gene co-expression network analysis
LASSO	Least absolute shrinkage and selection operator
SVM	Support vector machine
RF	Random forest
AUC	Area under the curve
GSEA	Gene set enrichment analysis
CD8	Cluster of differentiation 8
scTenifoldKnk	Single-cell tensor factorization for gene regulatory network inference
AI	Artificial intelligence
RDTs	Rapid diagnostic tests
DEGs	Differentially expressed genes
PCA	Principal component analysis
ROC	Receiver operating characteristic
CI	Confidence interval
*Pb*ANKA	*Plasmodium berghei* ANKA
IHC	Immunohistochemistry
DNA	Deoxyribonucleic acid
RNA	Ribonucleic acid
ceRNA	Competing endogenous RNA
miRNA	MicroRNA
lncRNA	Long non-coding RNA
UMAP	Uniform manifold approximation and projection
t-SNE	t-distributed stochastic neighbor embedding
ANX005	Anti-C1q antibody (C1q inhibitor)
H&E	Hematoxylin and eosin staining
TUNEL	Terminal deoxynucleotidyl transferase dUTP nick end labeling
DrugRefLector	AI-based drug prediction framework
HRP2	Histidine-rich protein 2
pLDH	*Plasmodium* lactate dehydrogenase
PCR	Polymerase chain reaction
LAMP	Loop-mediated isothermal amplification
C1QA	Complement component 1, q subcomponent, A chain
C1QB	Complement component 1, q subcomponent, B chain
C1QC	Complement component 1, q subcomponent, C chain
PU.1	Purine-rich box 1 (transcription factor)
IRF8	Interferon regulatory factor 8
C1q	Complement component 1q
GEO	Gene Expression Omnibus
GO	Gene Ontology
KEGG	Kyoto Encyclopedia of Genes and Genomes
MEs	Module eigengenes
SVM-RFE	Support Vector Machine–Recursive Feature Elimination
NES	Normalized enrichment score
mRNA	Messenger RNA
KO	Knockout
ICR	Institute of Cancer Research
ELISA	Enzyme-linked immunosorbent assay
RNA-seq	RNA sequencing
PBS	Phosphate-buffered saline
PFA	Paraformaldehyde
BSA	Bovine serum albumin
HRP	Horseradish peroxidase
DAB	3,3′-Diaminobenzidine
i.p.	Intraperitoneal (injection)
SSC	Saline-sodium citrate buffer
DAPI	4′,6-diamidino-2-phenylindole
PDB	Protein Data Bank
RCSB	Research Collaboratory for Structural Bioinformatics
ANOVA	Analysis of variance
