# Supplementary material for: C1QB-Mediated Immunopathology in a Murine Malaria Model: A Multi-Omics Validation for Diagnostic and Therapeutic Targeting
Source: Int J Mol Sci. 2026 Aug 20;27(16):7459. doi: 10.3390/ijms27167459 (PMC13513005; doi:10.3390/ijms27167459)
Supplement: Supplementary file 1 [file ijms-27-07459-s001.zip › Figure S1.pdf]

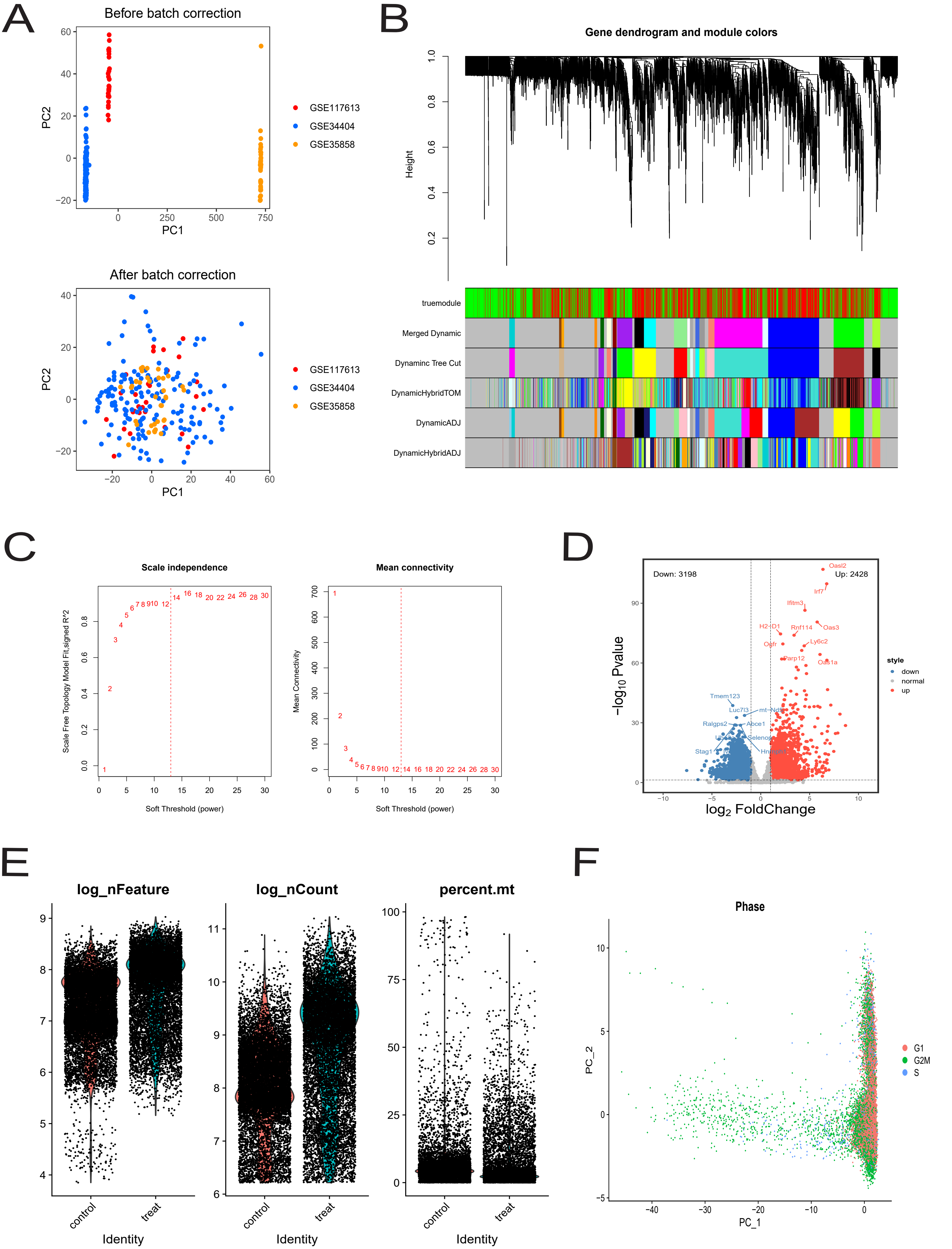
A
Before batch correction
PC1
PC2
GSE117613
GSE34404
GSE35858
After batch correction
B
Gene dendrogram and module colors
Height
truemodule
Merged Dynamic
Dynaminc Tree Cut
DynamicHybridTOM
DynamicADJ
DynamicHybridADJ
C
Scale independence
Scale Free Topology Model Fit,signed R^2
Soft Threshold (power)
Mean connectivity
Mean Connectivity
D
Down: 3198
Up: 2428
Oasl2
Irf7
Ifitm3
H2-D1
Rnf114
Oas3
Ly6c2
Parp12
Oas1a
Tmem123
Luc7l3
Ralgps2
Abce1
Stag1
style
down
normal
up
−log10 Pvalue
log2 FoldChange
E
log_nFeature
log_nCount
percent.mt
control
treat
Identity
F
Phase
PC_1
PC_2
G1
G2M
S
