# Supplementary material for: C1QB-Mediated Immunopathology in a Murine Malaria Model: A Multi-Omics Validation for Diagnostic and Therapeutic Targeting
Source: Int J Mol Sci. 2026 Aug 20;27(16):7459. doi: 10.3390/ijms27167459 (PMC13513005; doi:10.3390/ijms27167459)
Supplement: Supplementary file 1 [file ijms-27-07459-s001.zip › Figure S2.pdf]

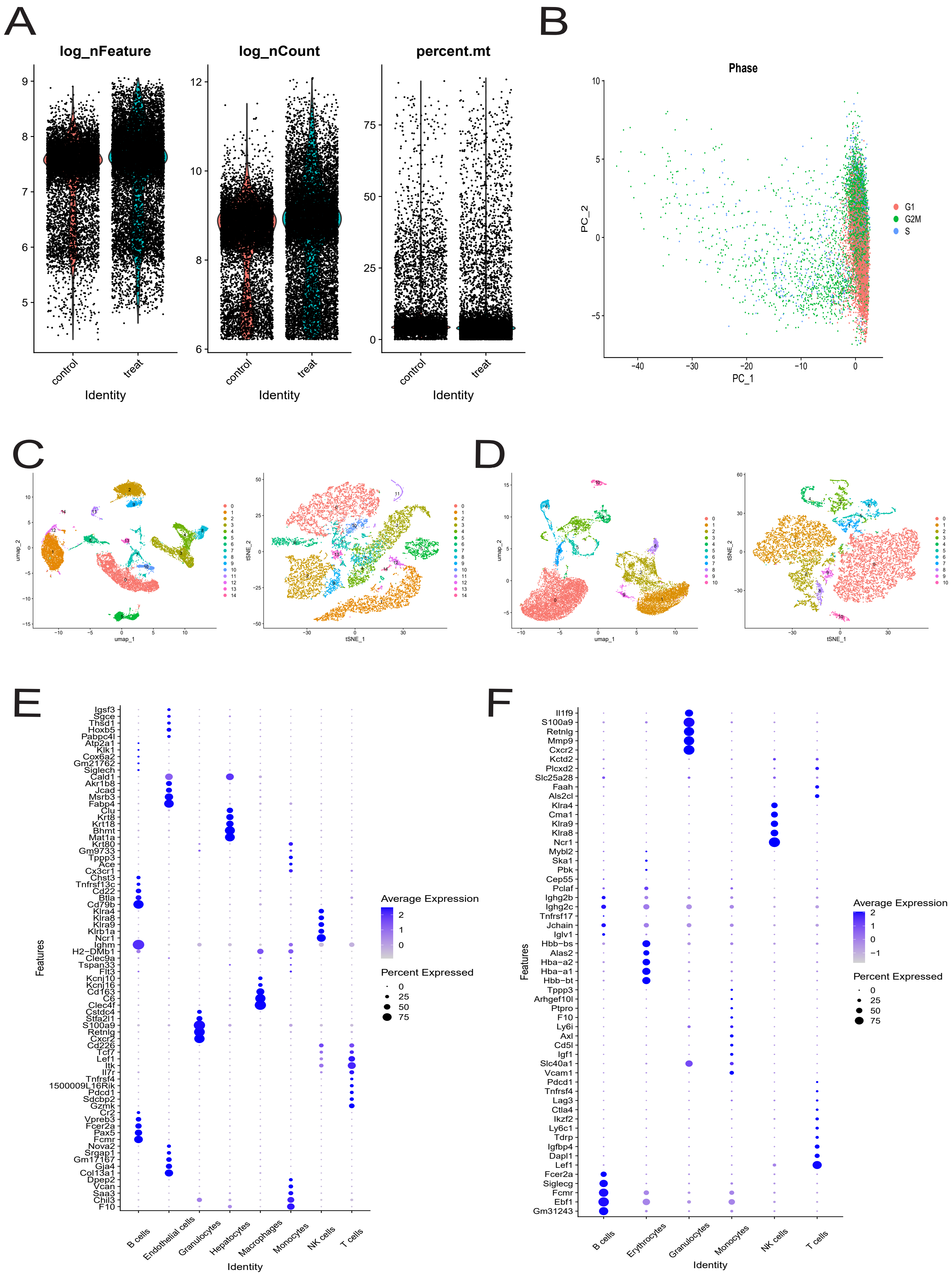
A
log_nFeature
log_nCount
percent.mt
control
treat
Identity
B
Phase
PC_2
PC_1
G1
G2M
S
C
umap_1
umap_2
tSNE_1
tSNE_2
D
E
Igsf3
Sgce
Thsd1
Hoxb5
Pabpc4l
Atp2a1
Klk1
Cox6a2
Gm21762
Siglech
Cald1
Akr1b8
Jcad
Msrb3
Fabp4
Clu
Krt8
Krt18
Bhmt
Mat1a
Krt80
Gm9733
Tppp3
Ace
Cx3cr1
Chst3
Tnfrsf13c
Cd22
Btla
Cd79b
Klra4
Klra8
Klra9
Klrb1a
Ncr1
Ighm
H2−DMb1
Clec9a
Tspan33
Flt3
Kcnj10
Kcnj16
Cd163
C6
Clec4f
Cstdc4
Stfa2l1
S100a9
Retnlg
Cxcr2
Cd226
Tcf7
Lef1
Itk
Il7r
Tnfrsf4
1500009L16Rik
Pdcd1
Sdcbp2
Gzmk
Cr2
Vpreb3
Fcer2a
Pax5
Fcmr
Nova2
Srgap1
Gm17167
Gja4
Col13a1
Dpep2
Vcan
Saa3
Chil3
F10
Features
B cells
Endothelial cells
Granulocytes
Hepatocytes
Macrophages
Monocytes
NK cells
T cells
Identity
Average Expression
Percent Expressed
F
Il1f9
S100a9
Retnlg
Mmp9
Cxcr2
Kctd2
Plcxd2
Slc25a28
Faah
Als2cl
Klra4
Cma1
Klra9
Klra8
Ncr1
Mybl2
Ska1
Pbk
Cep55
Pclaf
Ighg2b
Ighg2c
Tnfrsf17
Jchain
Iglv1
Hbb−bs
Alas2
Hba−a2
Hba−a1
Hbb−bt
Tppp3
Arhgef10l
Ptpro
F10
Ly6i
Axl
Cd5l
Igf1
Slc40a1
Vcam1
Pdcd1
Tnfrsf4
Lag3
Ctla4
Ikzf2
Ly6c1
Tdrp
Igfbp4
Dapl1
Lef1
Fcer2a
Siglecg
Fcmr
Ebf1
Gm31243
Features
B cells
Erythrocytes
Granulocytes
Monocytes
NK cells
T cells
Identity
Average Expression
Percent Expressed
