# Supplementary material for: C1QB-Mediated Immunopathology in a Murine Malaria Model: A Multi-Omics Validation for Diagnostic and Therapeutic Targeting
Source: Int J Mol Sci. 2026 Aug 20;27(16):7459. doi: 10.3390/ijms27167459 (PMC13513005; doi:10.3390/ijms27167459)
Supplement: Supplementary file 1 [file ijms-27-07459-s001.zip › Supplementary_file.pdf]

Figure S1

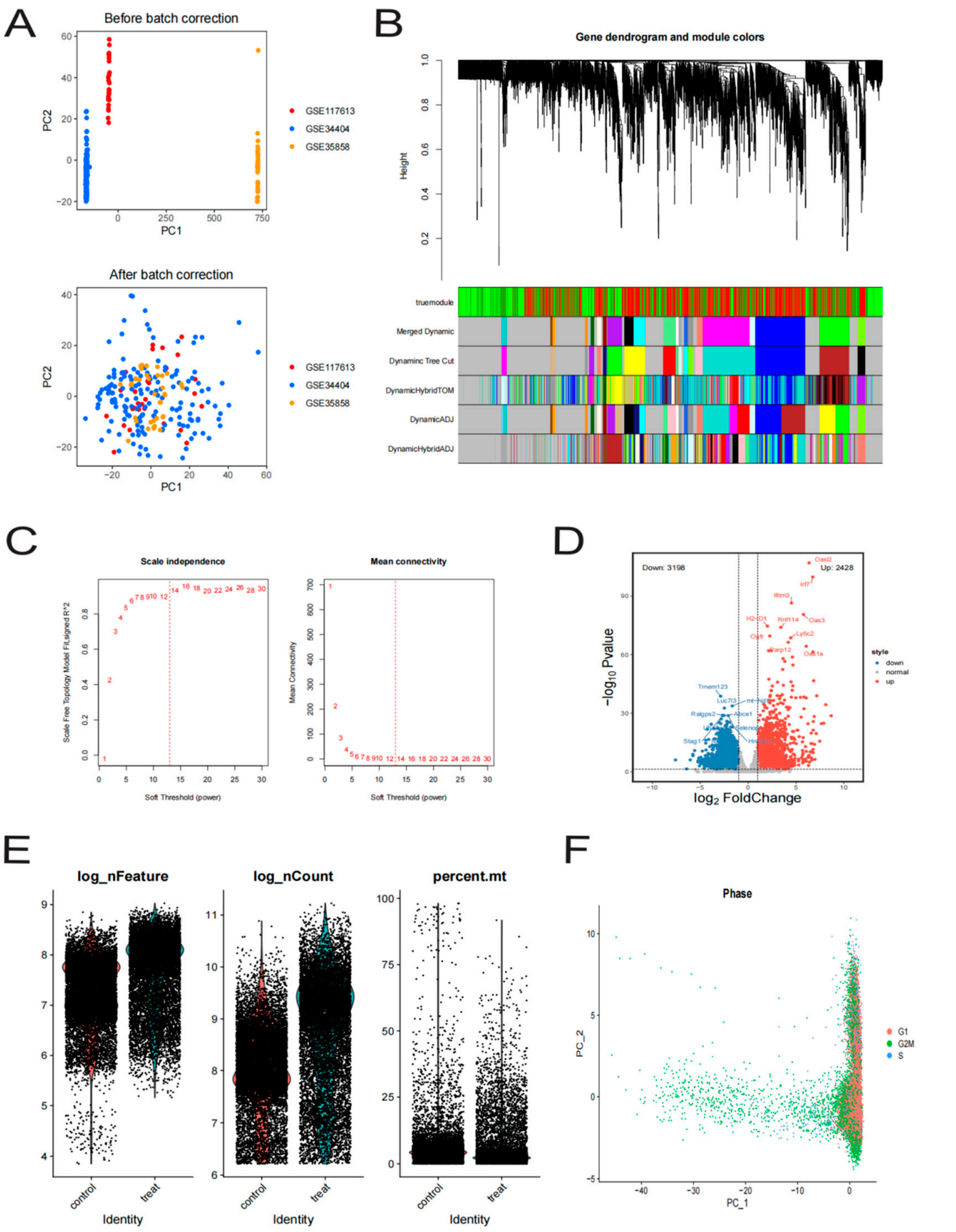

Figure S1. (A) PCA showed homogeneous clustering of the integrated samples after batch-effect correction. (B) Dendrogram of genes clustered by WGCNA, with different colors representing distinct gene modules. (C) Analysis of network topology to determine the soft-thresholding power. (D) Volcano plot of DEGs in peripheral blood of malaria-infected mice. (E) QC metrics of single-cell data in liver. (F) PCA analysis of cells based on cell cycle phase (G1, S, G2M) in liver.

Figure S2

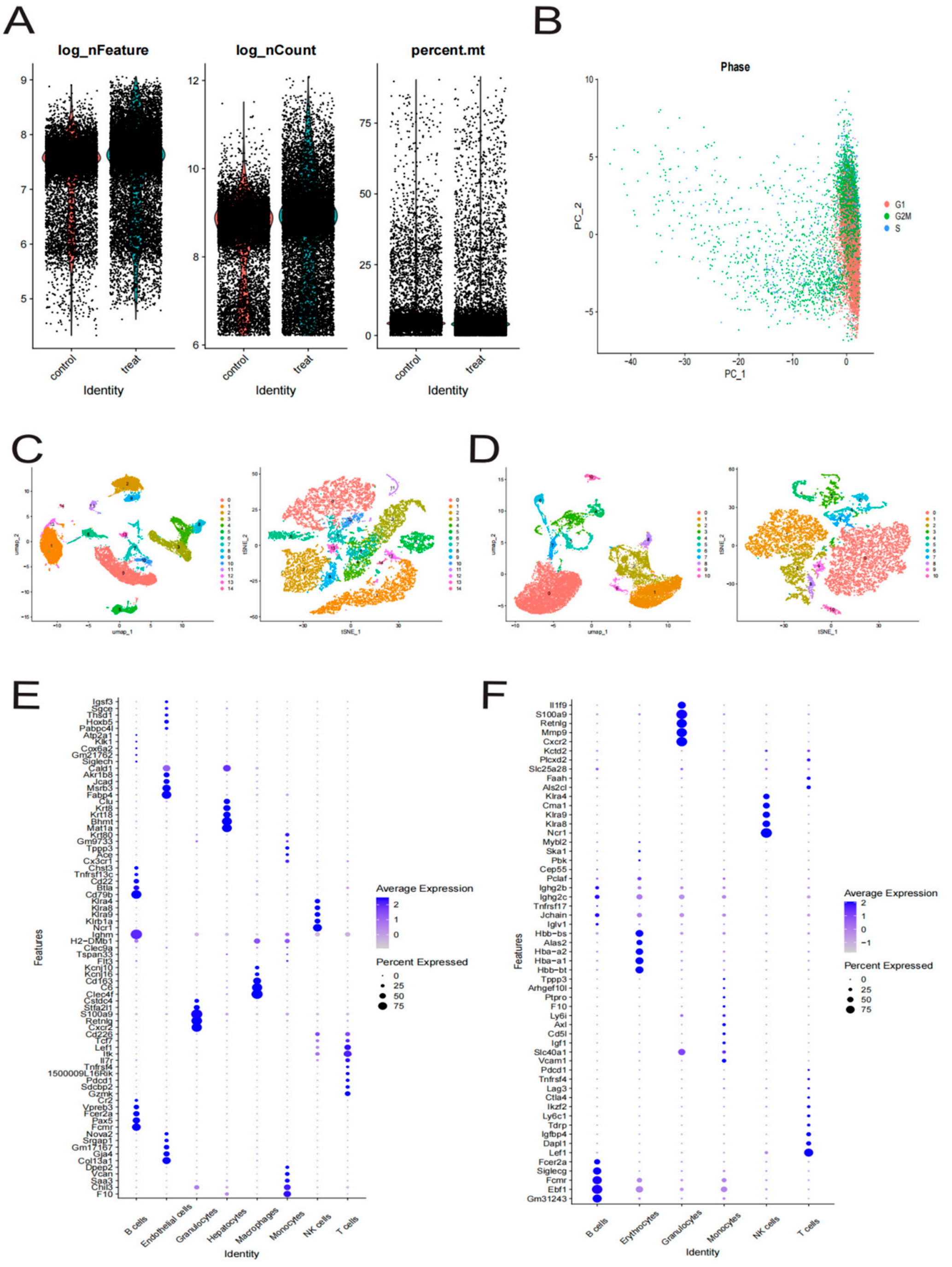

Figure S2. (A) QC metrics of single-cell data across samples in spleen. (B) PCA analysis of cells based on cell cycle phase (G1, S, G2M) in spleen. (C-D) Dimensionality reduction using t-SNE and UMAP, revealing distinct cell clusters. C, liver. D, spleen. (E) Bubble chart illustration of marker in different clusters. E, liver. F, spleen.
